# Synergistic Activity of Colistin/Fosfomycin Combination against Carbapenemase-Producing* Klebsiella pneumoniae* in an* In Vitro* Pharmacokinetic/Pharmacodynamic Model

**DOI:** 10.1155/2018/5720417

**Published:** 2018-04-23

**Authors:** Jin Wang, Ji-tong He, Yan Bai, Rui Wang, Yun Cai

**Affiliations:** Center of Medicine Clinical Research, Department of Pharmacy, PLA General Hospital, Beijing, China

## Abstract

Carbapenemase-producing* Klebsiella pneumoniae* is globally recognized as one of the greatest threats to public health, and combination therapy may be the chemotherapeutic option. In the present study, we aimed to evaluate the antibacterial effects of colistin/fosfomycin combination against carbapenemase-producing* K. pneumoniae*. The antibacterial effects were determined in a one-compartment* in vitro* pharmacokinetic model over a period of 24 h. The initial inoculum was 10^8^ CFU/mL. Low, medium, and high *C*_max_ values of colistin at 0.5, 2, and 5 mg/L as well as *C*_max_ of fosfomycin at 100 mg/L were simulated in the model. Doses of both colistin and fosfomycin were given every 8 h until 24 h. For the colistin- and fosfomycin-susceptible isolate KP47, three combination regimens showed greater killing effect compared with colistin monotherapy. The greatest killing effect was observed in combination regimen containing 5 mg/L colistin. For colistin-heteroresistant and fosfomycin-susceptible isolate KP79, combination regimen containing low dose colistin (0.5 mg/L) showed no synergistic or additive effects. However, combination regimens containing 2 and 5 mg/L colistin maintained the bactericidal effect until 24 h compared with colistin monotherapy. For colistin-heteroresistant and fosfomycin-resistant isolates KP42 and KP11, bactericidal activity was barely enhanced by combination regimens. Moreover, combination regimen containing 5 mg/L colistin could only prevent the emergence of colistin-resistant subpopulation in colistin and fosfomycin-susceptible isolate. It is necessary to know the resistant patterns of the* K. pneumoniae* before using combination of colistin and fosfomycin in clinical practice.

## 1. Introduction

The emergence of carbapenem-resistant Enterobacteriaceae (CRE), particularly carbapenemase-producing* Klebsiella pneumoniae*, has greatly increased over the past decade and become a significant public health concern as carbapenem is one of the few antibiotics to treat severe infections caused by these pathogens [1]. A recent survey from 36 countries in Europe about carbapenemase-producing Enterobacteriaceae, including more than 400 hospitals, has shown that 850 (37%) of 2,301* K. pneumoniae* samples are carbapenemase producers [2]. However, the discovery of new antibiotics cannot catch up with growth of antimicrobial resistance [3]. Prevalence of resistance coupled with scarcity of novel antimicrobials forces us to reevaluate some “old but efficacious” antibiotics. Colistin is one of these old drugs.

As a polypeptide antibiotic, colistin belongs to the polymyxin family. Colistin disrupts the outer cell membrane of the Gram-negative bacilli by binding to the lipid A component of the lipopolysaccharide, causing leakage of cytoplasmic contents and bacterial cell death [4]. An alarming increase in the rate of resistance to most available antimicrobials and a shortage of new antimicrobial agents have forced researchers to reevaluate the use of colistin, despite its potential risk of nephrotoxicity and neurotoxicity [5]. Colistin has been recently applied against the widespread multidrug-resistant (MDR) Gram-negative pathogens, including* K. pneumoniae.* Unfortunately, colistin resistance has also been reported in* K. pneumoniae *[6]. Heteroresistance observed in colistin monotherapy is considered to be a major cause of the rapid emergence of colistin resistance [7–10], which may be mitigated by combination therapy. In this case, the combination therapy becomes an option. Fosfomycin is another old broad-spectrum antibiotic that inhibits bacterial cell wall synthesis. Due to its excellent clinical efficacy and tolerability, fosfomycin has been successfully used in the treatment of uncomplicated urinary tract infections and pyelonephritis in females. From an* in vitro* standpoint, fosfomycin generally has high activity against ESBL- and carbapenemase-producing Enterobacteriaceae [11]. A study has showed that 78% of 107 carbapenem-nonsusceptible Enterobacteriaceae isolates remained susceptible to fosfomycin [12]. Our previous* in vitro* studies have confirmed that combination of colistin and fosfomycin shows synergy against carbapenem-resistant* Pseudomonas aeruginosa* isolates [13].

In the present study, we aimed to evaluate the antibacterial effects of colistin/fosfomycin combination against carbapenemase-producing* K. pneumoniae *with varying susceptibility to fosfomycin. An* in vitro* pharmacokinetic/pharmacodynamic (PK/PD) model was applied to simulate PKs of clinically achievable colistin and fosfomycin at routine doses in patients. The risk of emergence of secondary resistance was investigated by studies of population analysis profiles (PAPs).

## 2. Materials and Methods

### 2.1. Strains

Four clinical carbapenem-resistant* K. pneumoniae* isolates (KP47, KP79, KP11, and KP42) were selected according to different susceptibilities to colistin and fosfomycin. KP11 was collected from the Navy General Hospital; KP42 and KP47 were obtained from Beijing Hospital; and KP79 was isolated from the PLA General Hospital.

Minimum inhibitory concentration (MIC) of colistin was tested via broth (cation adjusted Mueller-Hinton) microdilution method, while MIC of fosfomycin was determined via agar (Mueller-Hinton agar with 25 *μ*g/mL glucose-6-phosphate, G6P) dilution method. CLSI epidemiological cutoff values of colistin and EUCAST breakpoint of fosfomycin for Enterobacteriaceae were employed. If the MIC of colistin against an isolate was ≤2 mg/L, while its subpopulations were able to survive on the plates containing colistin higher than 2 mg/L, such isolate would be defined as heteroresistance to colistin [14]. A biochemical test (Carba NP test II) was used to identify different types of carbapenemase production in* K. pneumoniae* (classes A, B, and D).

### 2.2. Antibiotics and Adjuvants

Colistin sulfate (Lot. WXBB6005V ≥ 15,000 U/mg) and G6P (Lot. 423A0313, 98–100%) were obtained from Sigma-Aldrich. Fosfomycin (Lot. 0350-200001, 65.1%) was purchased from the National Institutes for the Food and Drug Control (Beijing, China). Colistin sulfate was applied in this study because colistin methanesulfonate (CMS) is a prodrug that turns into its active form when administrated* in vivo *[15], although CMS is commonly used in clinical practice.

### 2.3. *K. pneumoniae* Genotyping

PCR was used to amplify the *β*-lactamase-associated genes. Supplementary Table S1 lists 15 sets of primers for *β*-lactamases of Ambler class A (SME, IMI, VEB, KPC, SHV, GES, and CTX), Ambler class B (NDM, VIM, and IMP), Ambler class C (AmpC), and Ambler class D (OXA-48, OXA-10, OXA-2, and OXA-1) [16]. Amplification procedure is applied as described in previous study with minor revision and suitable for 15 *β*-lactamase-associated genes listed above [17]. After an initial denaturation step at 94°C for 4 min, amplifications were carried out with 36 cycles at a melting temperature of 94°C for 1 min, an annealing temperature of 49°C for 1 min, and an extension temperature of 70°C for 5 min, followed by a final elongation step at 72°C for 10 min.

### 2.4. In Vitro PK/PD Model

**Figure 1:**
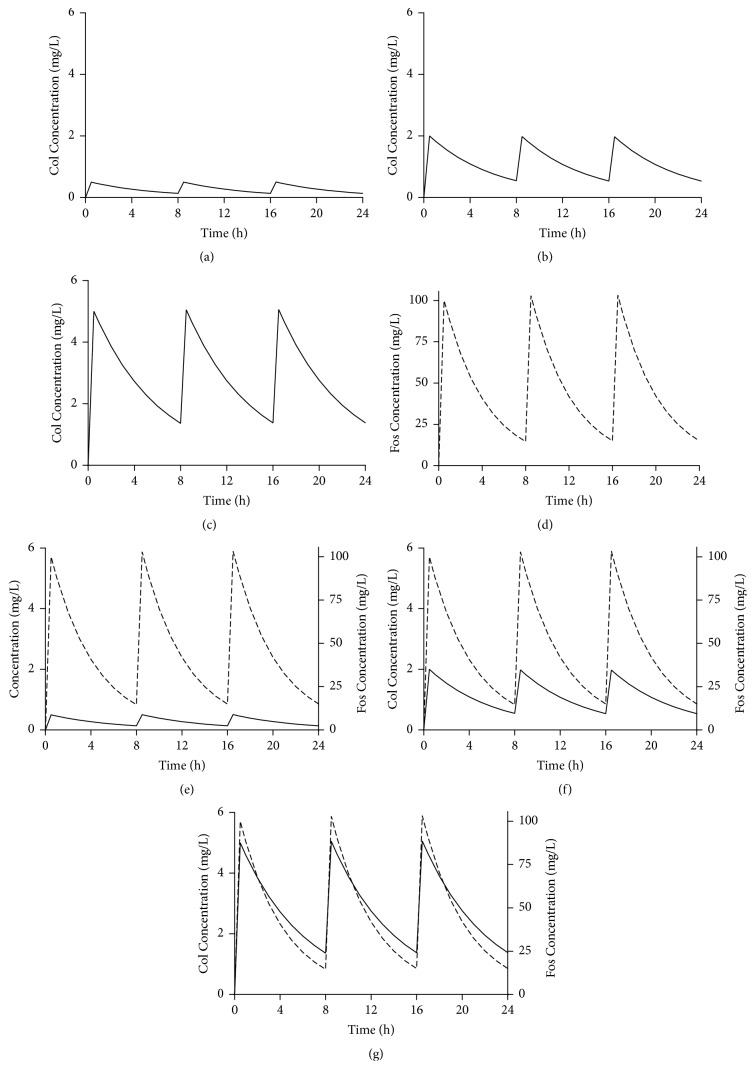
Targeted steady-state concentration-time profiles for colistin (Col, every 8 h) alone or in combination with fosfomycin (Fos, every 8 h). (a) Col 0.5 mg/L, (b) Col 2 mg/L, (c) Col 5 mg/L, (d) Fos 100 mg/L, (e) Col 0.5 mg/L plus Fos 100 mg/L, (f) Col 2 mg/L plus Fos 100 mg/L, and (g) Col 5 mg/L plus Fos 100 mg/L.

A one-compartment* in vitro* PK/PD model (PASS-402W System, Japan) was employed to evaluate the bactericidal effects of colistin alone or in combination with fosfomycin over a period of 24 h. The apparatus consisted of one central chamber with a capacity of 200 mL that was connected to a reservoir containing CAMHB broth. The temperature of broth within the system was maintained at 37°C. Fresh broth was pumped into the central chamber through a peristaltic pump at a defined flow rate. There was mixing to ensure distribution. Low, medium, and high *C*_max_ values of colistin at 0.5, 2, and 5 mg/L as well as *C*_max_ of fosfomycin at 100 mg/L were simulated in this PK/PD model. Simulated half-life for colistin and fosfomycin was 4 h and 2.7 h, respectively, as suggested in previously study [18]. Colistin concentrations were modified from the PK profiles in previously published report of* in vitro* PK/PD study, which maintained a constant concentration of colistin at 0.5, 2, or 5 mg/L [19]. Fosfomycin regimen was also simulated according to the PK parameters achieved in clinic [20]. At the beginning of the experiments, stock solutions of colistin and fosfomycin were injected into the model to achieve the target maximum concentration, and bolus injections were given every 8 h till 24 h. In addition, 25 mg/L of G6P was maintained in the MHB medium throughout the entire experiments.  Figure 1 illustrates the targeted steady-state concentration-time profiles of colistin alone or in combination with fosfomycin. In this PK/PD model, synergy is defined as a decrease of *⩾*2 log⁡10 in the number of CFU/mL between the combination and its most active component; additivity is defined as a decrease of 1.0 to <2 log⁡10 in the number of CFU/mL between the combination and its most active component [21].

### 2.5. Bactericidal Effects and Emergence of Colistin Resistance

Briefly, 1 mL of serial samples was drawn from the central chamber at defined time points listed in Table 2 for viable colony count and real-time PAPs. Viable colony count and PAPs were conducted right after sampling. Subsequently, 50 *μ*L of each sample with serial dilution was spirally plated on agar plates. Viable colony count was conducted after 24 h incubation at 35°C.

## 3. Results

### 3.1. Characteristics of* K. pneumoniae* Isolates

All four strains were confirmed as producers of class A carbapenemases by Carba NP test.  Table 1 shows the results of MICs and *β*-lactamase genotyping. The four strains were all carbapenem-resistant with MICs of imipenem and meropenem ≥ 128 mg/L. MIC of colistin against four strains were ≤1 mg/L. However, except for KP47, the other three strains were colistin-heteroresistant because their subpopulations could grow on the nutrient agar with colistin concentration > 2 mg/L. KP47 and KP79 were susceptible to fosfomycin with the MICs of 1 and 16 mg/L, respectively. KP11 and KP42 were resistant to fosfomycin with the MIC > 256 mg/L.

### 3.2. Antibacterial Effects

#### 3.2.1. Colistin Monotherapy

**Figure 2:**
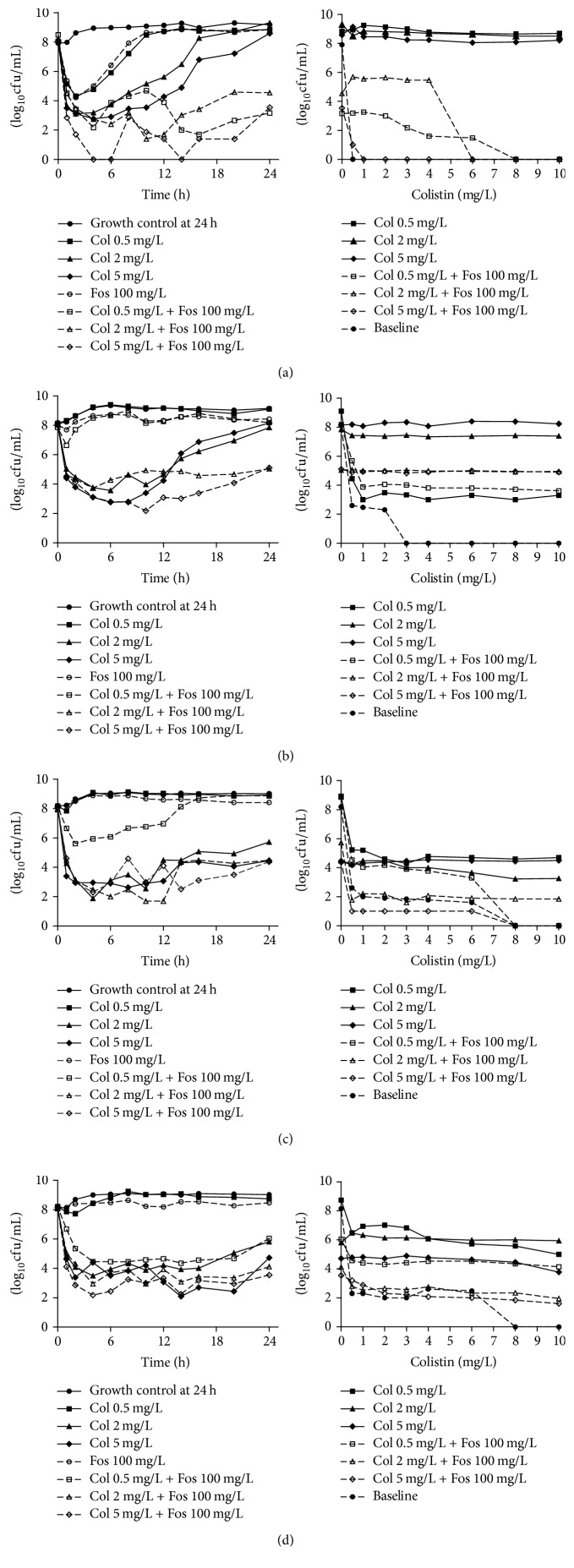
(Left) time-kill curves with various clinically relevant dosage regimens of colistin (Col) and fosfomycin (Fos) alone and in combination at an inoculum of 10^8^ CFU/mL. (Right) PAPs at baseline and after 24 h of exposure to colistin monotherapy, colistin-fosfomycin combination therapy, or neither antibiotic (growth control). Fosfomycin monotherapy regimens were not included in colistin-PAP examination. (a) KP47 (colistin-susceptible, fosfomycin-susceptible); (b) KP79 (colistin-heteroresistant, fosfomycin-susceptible); (c) KP42 (colistin-heteroresistant, fosfomycin-resistant); (d) KP11 (colistin-heteroresistant, fosfomycin-resistant).

Colistin monotherapy with low, medium, and high* C*max showed different bacterial killing effects against colistin-susceptible and colistin-heteroresistant* K. pneumoniae* strains. For the colistin-susceptible isolate KP47, colistin alone produced rapid initial (0–4 h) killing effect to 3 log⁡10 CFU/mL or below with all three concentrations of colistin. The regrowth of bacteria to the level of the control group occurred at 10 h, 16 h, and 24 h with colistin monotherapy at 0.5, 2, and 5 mg/L, respectively (Figure 2(a), Table 3). For the colistin-heteroresistant isolates KP79, KP42, and KP11, 0.5 mg/L colistin alone essentially produced no bacterial killing effect against any isolate (Figures 2(b), 2(c), and 2(d), Table 3). Colistin at 2 and 5 mg/L caused a reduction about 4 log⁡10 CFU/mL against KP79 till 10 h, and both regrowth to the level of the control group occurred at 24 h (Figure 2(b), Table 3). For KP42 and KP11, which were heteroresistant to colistin and resistant to fosfomycin, colistin monotherapy at 2 and 5 mg/L achieved greatest bactericidal effect by 10 h (4-5 log⁡10 CFU/mL), and the effect remained to 24 h (2-3 log⁡10 CFU/mL) compared with the control (Figures 2(c) and 2(d), Table 3). In general, greater bactericidal effects were observed in colistin regimen with higher targeted *C*max/MIC. *C*max/MIC of colistin at 5 mg/L against KP42 and KP11, which were higher than other regimen (Table 2), exerted consistent bactericidal effect throughout the 24 h (Table 3).

#### 3.2.2. Fosfomycin Monotherapy

Fosfomycin monotherapy was conducted at a *C*_max_ of 100 mg/L. Except for colistin-susceptible isolate KP47, a maximum reduction around 3 log⁡10 CFU/mL was observed at 2 h, and then it started to regrowth. Fosfomycin showed little bacterial killing effect against three colistin-heteroresistant isolates no matter they were resistant or susceptible to fosfomycin.

#### 3.2.3. Combination Therapy

Enhanced bacterial killing effect was particularly evident for the colistin- and fosfomycin-susceptible isolate KP47 (Figure 2(a) and Table 3). A combination therapy of fosfomycin (at 100 mg/L *C*max) and colistin (at 0.5 mg/L *C*max) produced a 3-log⁡10 higher killing effect at 4 h posttreatment compared with the monotherapy. Even at 24 h, combination of fosfomycin and colistin still showed a greater killing effect compared with colistin monotherapy. Combination of fosfomycin and 2 mg/L colistin produced more than 4 log⁡10 greater killing effect compared with colistin monotherapy at 10–24 h. The greatest killing effect was observed in combination regimen containing 5 mg/L colistin. Bacterial killing below the limit of detection sustained from 2 h to 20 h compared with colistin alone. Of the 18 cases from 4–24 h (three combinations across six time points), 15 cases showed synergistic effect, and one case exhibited additive effect (Table 3).

For colistin-heteroresistant and fosfomycin-susceptible isolate KP79, combination of low dose colistin (0.5 mg/L) and fosfomycin showed no synergistic or additive effects compared with colistin alone (Figure 2(b) and Table 3). Moreover, combination of 2 or 5 mg/L colistin and fosfomycin exhibited similar bactericidal effect with colistin monotherapy before 12 h. However, regrowth started after 12 h in colistin monotherapy and almost reached the original inoculum at 24 h, while the combination therapy maintained the bactericidal effect until 24 h. In addition, five cases of synergistic effect and two cases of additive effect were observed in the eight cases from 12–24 h (two combinations across four time points, Table 3).

For colistin-heteroresistant and fosfomycin-resistant isolates KP42 and KP11, enhanced activity was only noticeable in the combination of low dose colistin (0.5 mg/L) and fosfomycin. This combination yielded additional bactericidal effect of more than 2 log⁡10 before 12 h against KP42. After 12 h, its regrowth had no significant difference with low dose colistin alone. For KP11, combination of 0.5 mg/L colistin and fosfomycin sustained a 3-4 log⁡10 CFU lower through 24 h compared with colistin alone. There was no enhancement of bactericidal activity in combination containing 2 or 5 mg/L colistin, showing similar efficacy compared with colistin alone, except that combination resulted in greater activity with additive (predominantly) or synergistic effect at 3-4 time points.

### 3.3. Emergence of Colistin Resistance

For KP47, the colistin-susceptible strain, colistin monotherapy with *C*_max_ at 0.5, 2, or 5 mg/L at inoculum of 10^8^ CFU/mL could increase the growth of colistin-resistant subpopulations at 24 h. These subpopulations could grow well on the plates containing 10 mg/L colistin (Figure 2(a)). Combinations of low or medium dose of colistin and fosfomycin did not prevent the emergence of colistin-resistant isolates against KP47. The subpopulation could still grow in the presence of 4 or 6 mg/L colistin. However, combination containing 5 mg/L colistin could completely inhibit the emergence of colistin-resistant subpopulations. Not even a single colony could be detected on plates with the concentration of colistin ≥ 1 mg/L by 24 h (Figure 2(a)). However, for colistin-heteroresistant isolates (KP79, KP42, and KP11), no matter they were fosfomycin-susceptible or fosfomycin-resistant, all combinations of colistin and fosfomycin at inoculum of 10^8^ CFU/mL did not eliminate the colistin-resistant subpopulations.

## 4. Discussion

Colistin has been rekindled as the last resort therapeutic regimen due to the increasing prevalence of MDR Gram-negative pathogens worldwide. However, colistin-resistant species, such as Enterobacteriaceae,* Pseudomonas *spp., and* Acinetobacter *spp., have also been reported since colistin is more and more used in the treatment of MDR pathogen-caused infections. It has been reported that the occurrence of colistin resistance may be due to suboptimal use of colistin, such as suboptimal dose or prolonged monotherapy [22, 23]. Even for those pathogens that are initially susceptible to colistin, regrowth can be observed after the colistin monotherapy [21, 24]. In our study, colistin monotherapy induced the colistin resistance against the initially colistin-susceptible isolate KP47 within 24 h. Combination therapy has become an alternative to improve effectiveness and prevent antimicrobial resistance due to the toxicity limiting dose escalation of colistin.

In the present study, we evaluated the antimicrobial effects of colistin/fosfomycin combination against carbapenemase-producing* K. pneumoniae* in terms of their synergistic activity and ability of preventing emergence of resistant subpopulations. The concentrations of colistin and fosfomycin were selected based on clinically achievable serum free drug concentrations at commonly recommended dosages. The package insert of CMS in the USA recommends that dose of intravenous injection ranges from 2.5 mg/kg to 5 mg/kg per day, which can be divided into two to four equal doses. *C*_max_ of formed colistin can reach 5 mg/L at steady state [25]. Generally speaking, total daily intravenous fosfomycin doses in patients range from 12 to 16 g per day, which can be divided into two-four equal doses. High doses of fosfomycin (up to 24 g) can also be given to patients with central nervous system infections or other severe infections, which will lead to very high maximal plasma concentrations of more than 300 mg/L [26].

Several studies have evaluated the* in vitro* and* in vivo* synergism of colistin/fosfomycin combination against Gram-negative bacteria. Wei et al. [27] have found that colistin in combination with fosfomycin shows synergistic effect against 50% of extensively drug-resistant (XDR)* A. baumannii*. Another clinical trial of 94 patients infected with carbapenem-resistant* A. baumannii* has demonstrated that those receiving colistin combination regimens show a significantly more favorable microbiological response and lower mortality compared with those receiving colistin monotherapy [28]. For carbapenem-resistant* Pseudomonas aeruginosa*, combination of colistin and fosfomycin exerts synergistic or partial synergistic effect against 21.84% or 27.59% of 87 isolates, while antagonism is not observed [13]. Combination of colistin and fosfomycin can prevent regrowth of extended-spectrum *β*-lactamase-producing (ESBL)* Escherichia coli* at a subinhibitory concentration. This combination also demonstrates the highest cure rate (67%) in a mice model of foreign-body infection, showing a much better effect compared with colistin or fosfomycin alone [29].

Previous study has also reported that colistin in combination with fosfomycin shows synergistic effect against* Klebsiella pneumoniae* carbapenemase- (KPC-) producing or metallo-*β*-lactamase- (MBL-) producing* K. pneumoniae*. In a time-kill study, Souli et al. have investigated the bactericidal effect of colistin/fosfomycin combination against 17 clinical* K. pneumoniae* isolates carrying blaKPC-2. This combination shows bactericidal activity against 11 (64.7%) and synergistic effect against two (11.8%) of the 17 isolates [30]. Tängdén et al. [31] have found that combination of colistin and fosfomycin exhibits synergistic and bactericidal effect against three of four MBL-producing* K. pneumoniae* strains (one VIM-KP and two NDM-KP). All of the four strains are susceptible to colistin, while two VIM-KP strains are fosfomycin-susceptible and two NDM-KP strains are fosfomycin-resistant. A clinical research has been performed in 11 ICUs including 48 cases. KPC-2-producing CRKP is isolated from 41 patients (85.4%), as either the single pathogen (23 of 48 cases; 47.9%) or mixed with other pathogens (18 of 48 cases; 37.5%). Colistin or tigecycline is the most common agent administered with fosfomycin. The results have shown that 60% patients with CRKP infections have the satisfactory clinical and microbiological outcomes [32]. Albur et al. [18] have applied a PK/PD model to evaluate the bactericidal effect of combination of colistin (*C*_max_ 3 mg/L) and fosfomycin (*C*_max_ 250 mg/L) against six NDM-1-producing Enterobacteriaceae isolates, including two colistin-susceptible* K. pneumoniae*. They have found that the combination regimen can increase bactericidal effect and reduce emergence of colistin resistance compared with each monotherapy. Moreover, the antibacterial effects of this combination can last for a longer time and are notable for both fosfomycin-susceptible and fosfomycin-resistant isolates. A recent study in 2017 has evaluated the bactericidal effect of colistin/fosfomycin combination against two KPC-producing* K. pneumoniae* stains, one of which is susceptible to both colistin and fosfomycin, while the other one is resistant to colistin and susceptible to fosfomycin.* In vitro* time-kill experiments have revealed that only highest concentrations of colistin (4 mg/L) and fosfomycin (512 mg/L) can achieve synergism in colistin-resistant KPC strains, while synergy is observed for all colistin/fosfomycin combinations against the double-susceptible KPC strain. They have only applied double-susceptible KPC strain in the subsequent PK/PD study. The results have shown that colistin or fosfomycin monotherapy results in rapid proliferation of resistant subpopulations. However, combination of colistin and fosfomycin leads to a rapid reduction of the double-susceptible KPC strain in 6 h and complete suppression of resistant subpopulations until 120 h [33]. Our results on the colistin-susceptible* K. pneumoniae* isolate were consistent with these reports. The antibacterial effects were enhanced after colistin was used in combination with fosfomycin against KP47, which is susceptible to both colistin and fosfomycin. The highest dose combination (*C*_max_ of 5 mg/L colistin and 100 mg/L fosfomycin) could completely prevent the emergence of colistin-resistant subpopulations. Because the concentration of fosfomycin applied in our study is lower than the above 2 studies (100 mg/L versus 250 and 300 mg/L), we speculated that if higher concentration of fosfomycin was included, more obvious synergism might be observed even in those colistin-heteroresistant strains.

However, the antimicrobial efficacy of the combination of colistin and fosfomycin was not obvious against the colistin-heteroresistant* K. pneumoniae*, even with the highest dose regimen, although the combination regimen could delay the regrowth of bacteria to a certain extent. Previous PK/PD research on combination of colistin (0.5 or 2 mg/L) and doripenem (*C*_max_ of 2.5 or 25 mg/L over 8 h) at constant concentrations against* K. pneumoniae* has shown that this combination can both enhance the bactericidal effect and prevent the occurrence of the colistin-resistant colonies at an inoculum of either 10^6^ or 10^8^ CFU/mL [21]. In contrast to their study, constant concentration of colistin was not applied in our study, which might explain the difference between these two studies. However, in general, the combination of colistin and fosfomycin was less effective compared with doripenem in the case of colistin-heteroresistant* K. pneumoniae*.

There are three limitations in the present study. Colistin and fosfomycin concentrations in CAMHB broth were only measured before experiment. The results showed that the actual concentrations were close to the predicted concentrations with variability of less than 5%. However, the actual concentration of either colistin or fosfomycin was not measured during the real experiments. The second limitation is that the bactericidal effect of combination was only examined up to 24 h. Therefore, we have no idea about resistance and regrowth after 24 h. The third is that only 4* K. pneumoniae* strains were applied in the present study, so the representativeness of the results needs to be further verified in more* K. pneumoniae *isolates and other species.

In conclusion, our study showed that combination of colistin and fosfomycin at clinically achievable concentrations could increase the bactericidal effects against colistin-susceptible* K. pneumoniae* and prevent the emergence of colistin-resistant subpopulations. However, this synergism and resistance preventing ability was barely observed in colistin-heteroresistant* K. pneumoniae*, especially in those fosfomycin-resistant isolates. Considering that colistin heteroresistance and fosfomycin resistance are not rare, it is necessary to know the resistant patterns of the* K. pneumoniae* when using combination of colistin and fosfomycin in clinical practice.

## Figures and Tables

**Table 1:** MICs for *K. pneumoniae *isolates used in this study.

Isolate	MIC (mg/L)		*β*-Lactamase genotyping	CarbaNP test	MLST	Colistin-heteroresistant^a^
	Colistin	Fosfomycin				
KP47	0.5	1	KPC, SHV, OXA-2, OXA-10	Ambler A	ST11	No
KP79	1	16	KPC, CTX, SHV, OXA-1, OXA-2, OXA-10	Ambler A	ST37	Yes
KP42	0.25	>256	KPC, CTX, SHV, OXA-10	Ambler A	ST11	Yes
KP11	0.125	>256	KPC, CTX, SHV, OXA-2, OXA-10	Ambler A	ST11	Yes

^a^Heteroresistance to colistin was defined as the existence, in an isolate for which the colistin MIC was ≤2 mg/L, of subpopulations able to grow in the presence of >2 mg/L colistin.

**Table 2:** Colistin and fosfomycin dosage regimens, PK/PD index values, and sampling times in the *in vitro* PK/PD model.

Treatment regimen	PK/PD index value												Sampling times (h) for microbiological measurements
	*f*AUC/MIC				*fC*max/MIC				*fT* > MIC				
	Kp47	Kp79	Kp42	Kp11	Kp47	Kp79	Kp42	Kp11	Kp47	Kp79	Kp42	Kp11	
*Colistin monotherapy, target concentration C*_*max*_ */C*_*min*_ * (mg/L)*													0, 1, 2, 4, 6, 8, 10, 12, 14, 16, 20, 24
0.5/0.14	11.38	5.69	22.76	45.52	1	0.5	2	4	0	0	100	100	
2/0.54	44.92	22.46	89.84	179.68	4	2	8	16	100	18.75	100	100	
5/1.36	113.82	56.91	227.64	455.28	10	5	20	40	100	100	100	100	

*Fosfomycin monotherapy, target concentration C*_*max*_ */C*_*min*_ * (mg/L)*													0, 1, 2, 4, 6, 8, 10, 12, 14, 16, 20, 24
100/14.58	837.26	52.33	<3.27	<3.27	100	6.25	<0.39	<0.39	100	100	0	0	

*Combination therapy*													0, 1, 2, 4, 6, 8, 10, 12, 14, 16, 20, 24

**Table 3:** Log changes at 4, 8, 12, 16, 20, and 24 h at an inoculum of 10^8^ CFU/mL with colistin and/or fosfomycin against *K. pneumoniae*.

Isolate	Time (h)	log change [log_10_(CFU_t_) − log_10_(CFU_0_)]						
		Col 0.5 mg/L	Col 2 mg/L	Col 5 mg/L	Fos 100 mg/L	Col 0.5 mg/L + Fos 100 mg/L	Col 2 mg/L + Fos 100 mg/L	Col 5 mg/L + Fos 100 mg/L
Kp47	4	*−3.23*	*−5.00*	*−5.24*	*−3.00*	**−6.30**	*−5.30*	**−8.13**
	8	−0.80	*−3.63*	*−4.57*	−0.08	**−4.17**	***−4.87***	*−5.27*
	12	0.73	*−2.58*	*−3.75*	0.70	**−4.60**	**−6.34**	**−6.73**
	16	0.74	0.08	*−1.23*	0.87	**−6.78**	**−4.62**	**−8.13**
	20	0.78	0.52	−0.81	0.66	**−5.83**	**−3.46**	**−6.73**
	24	0.83	1.11	0.56	0.84	**−5.32**	**−3.50**	**−4.59**

Kp79	4	1.10	*−4.29*	*−5.07*	0.60	0.63	*−4.32*	*−4.97*
	8	1.16	*−3.42*	*−5.38*	0.66	1.16	*−3.43*	*−5.33*
	12	1.03	*−3.40*	*−3.94*	0.27	0.41	*−3.19*	***−5.01***
	16	0.85	*−1.83*	*−1.30*	0.55	0.94	***−3.46***	**−4.71**
	20	0.66	*−1.10*	−0.69	0.31	0.58	**−3.37**	**−4.01**
	24	0.97	−0.21	−0.01	0.38	0.32	**−3.00**	**−2.97**

Kp42	4	0.93	*−6.14*	*−5.20*	0.79	**−2.20**	*−5.44*	*−5.80*
	8	0.97	*−4.53*	*−5.53*	0.78	**−1.47**	*−5.44*	*−3.51*
	12	0.87	*−3.52*	*−5.10*	0.50	**−1.18**	**−6.22**	*−4*
	16	0.81	*−2.94*	*−3.80*	0.47	0.58	*−3.43*	***−4.98***
	20	0.69	*−3.08*	*−4.10*	0.31	0.75	*−3.65*	*−4.60*
	24	0.76	*−2.29*	*−3.70*	0.32	0.71	***−3.44***	*−3.72*

Kp11	4	0.26	*−4.58*	*−3.79*	0.24	**−3.76**	*−5.15*	**−5.99**
	8	1.07	*−3.73*	*−4.35*	0.46	**−3.77**	*−4.17*	*−4.92*
	12	0.87	*−3.85*	*−5.12*	0.01	**−3.57**	*−4.16*	*−4.83*
	16	0.70	*−4.06*	*−5.48*	0.36	**−3.66**	*−4.65*	*− 5*
	20	0.67	*−3.02*	*−5.74*	0.10	**−3.54**	***−4.74***	*−5.21*
	24	0.56	*−2.26*	*−3.46*	0.29	**−2.18**	***−3.97***	***−4.62***

The italic font indicates activity (a reduction of ≥1 log_10_CFU/mL below the initial inoculum); bold font indicates synergy (a decrease of ≥2 log_10_ in the number of CFU/mL between the combination and its most active component); bold italic font indicates additivity (a decrease of 1.0 to <2 log_10_ in the number of CFU/mL between the combination and its most active component).

## Supplementary Materials

Supplementary Materials Primer sets for *β*-lactamases used in the PCR were listed as Table S1.
