# Supplementary material for: Synergistic Activity of Colistin/Fosfomycin Combination against Carbapenemase-Producing Klebsiella pneumoniae in an In Vitro Pharmacokinetic/Pharmacodynamic Model
Source: Biomed Res Int. 2018 Apr 23;2018:5720417. doi: 10.1155/2018/5720417 (PMC5937563; doi:10.1155/2018/5720417)
Supplement: Supplementary Materials — Primer sets for β-lactamases used in the PCR were listed as Table S1. [file 5720417.f1.pdf]

TABLE S1 Primer sets for β-lactamases used in the PCR.

Genes	Sequence of primer
bla-IMP-F	CTACCGCAGCAGAGTCTTTG
bla-IMP-R	AACCAGTTTTGCCTTACCAT
bla-VIM-F	AGTGGTGAGTATCCGACAG
bla-VIM-R	ATGAAAGTGCGTGGAGAC
bla-SME-F	AACGGCTTCATTTTTGTTTAG
bla-SME-R	GCTTCCGCAATAGTTTTATCA
bla-KPC-F	TGTCACTGTATCGCCGTC
bla-KPC-R	CTCAGTGCTCTACAGAAAACC
bla-IMI-F	CCATTCACCCATCACAAC
bla-IMI-R	CTACCGCATAATCATTTGC
bla-SHV-F	TGCGCAAGCTGCTGACCAGC
bla-SHV-R	TTAGCGYTGCCAGTGCTCGA
bla-GES-F	ATGCGCTTCATTCACGCAC
bla-GES-R	CTATTTGTCCGTGCTCAGG
bla-VEB-F	GCGGTAATTTAACCAGA
bla-VEB-R	GCCTATGAGCCAGTGTT
bla-OXA-1F	CTGTTGTTTGGGTTTCGCAAG
bla-OXA-1R	CTTGGCTTTTATGCTTGATG
bla-OXA-2F	CAGGCGCYGTTCGYGATGAGTT
bla-OXA-2R	GCCYTCTATCCAGTAATCGCC
bla-OXA-10F	GTCTTTCRAGTACGGCATTA
bla-OXA-10R	GATTTTCTTAGCGGCAACTTA
bla-OXA-48F	ATGCGTGTATTAGCCTTATCGGCT
bla-OXA-48R	CTAGGGAATAATTTTTTCCTGTTTGAG
bla-NDM-F	GCCAGCTCGCACCGAAT
bla-NDM-R	GAACGCCGCACCAAACG
bla-CTX-F	CGYTTTSCRATGTGCAG
bla-CTX-R	ACCGCRATATCRTTGGT
bla-AmpC1	ATGATGAAAAAATCGTTATGC
bla-AmpC2	TTGCAGCTTTTCAAGAATGCGC
